# Current Aspects of siRNA Bioconjugate for In Vitro and In Vivo Delivery

**DOI:** 10.3390/molecules24122211

**Published:** 2019-06-13

**Authors:** Wanyi Tai

**Affiliations:** Key Laboratory of Combinatorial Biosynthesis and Drug Discovery (Wuhan University), Ministry of Education, School of Pharmaceutical Sciences, Wuhan University, Wuhan 430071, Hubei, China; wanyi-tai@whu.edu.cn; Tel.: +86-027-68759987

**Keywords:** siRNA bioconjugate, siRNA scaffold, asymmetric siRNA, lipid siRNA conjugate, spherical siRNA

## Abstract

Studies on siRNA delivery have seen intense growth in the past decades since siRNA has emerged as a new class of gene therapeutics for the treatment of various diseases. siRNA bioconjugate, as one of the major delivery strategies, offers the potential to enhance and broaden pharmacological properties of siRNA, while minimizing the heterogeneity and stability-correlated toxicology. This review summarizes the recent developments of siRNA bioconjugate, including the conjugation with antibody, peptide, aptamer, small chemical, lipidoid, cell-penetrating peptide polymer, and nanoparticle. These siRNA bioconjugate, either administrated alone or formulated with other agents, could significantly improve pharmacokinetic behavior, enhance the biological half-life, and increase the targetability while maintaining sufficient gene silencing activity, with a concomitant improvement of the therapeutic outcomes and diminishment of adverse effects. This review emphasizes the delivery application of these siRNA bioconjugates, especially the conjugation strategy that control the integrity, stability and release of siRNA bioconjugates. The limitations conferred by these conjugation strategies have also been covered.

## 1. Introduction

RNA interference (RNAi) is a powerful gene silencing tool that utilizes 21–25-nucleotide (nt) small interfering RNA (siRNA) to degrade a particular mRNA in highly sequence-specific manner. siRNA has been considered to be the major player of gene therapy and shows broad therapeutic potential to treat a various gene-promoted diseases including viral infection and cancer [1]. Compared to chemical drugs and antibodies, it is a new class of drugs that is distinguished by targeting mRNA directly, thus suppressing the expression of the disease-causing proteins. Despite the huge therapeutic potential of siRNA, its clinical application remains challenging, mainly due to the lack of efficient delivery system [2,3]. siRNA has been under clinical development for approximately 16 years. During that lengthy period of time, dozens of siRNA have been evaluated in clinical trials, but only one siRNA-based drug (Patisiran) has been approved by the Food and Drug Administration (FDA) under orphan drug status [4,5]. In the preclinical studies, the siRNA delivery strategy has moved away from the complicate lipid or polymer nanoparticles that contain multicomponents for functionalities to the chemically well-defined siRNA bioconjugates that possess a more homogenous, stable, and biocompatible structure; a rationale bolstered by the successes of cholesterol siRNA conjugate (chol-siRNA) and GalNAc-siRNA conjugate [6,7,8]. 

Recently, bioconjugation has evolved as a powerful delivery strategy to confer siRNA a variety of functionalities for efficient delivery. It has been reported that siRNA can be linked with targeting ligands for cell type-specific delivery, lipids for better pharmacokinetics, cell-penetrating peptides (CPP) for cell uptake and endosome escape, and magnet nanoparticles for imaging and diagnosis. Compared to the unconjugated siRNA, such siRNA bioconjugate shows significantly higher tissue bioavailability and targetability, longer biological half-life and better pharmacological properties, with a concomitant increase of delivery efficiency while maintaining the gene silencing potency. In this review, we focus on the current aspects of various siRNA conjugate delivery systems that have potential to open new avenues for clinical utility. Advances in chemical conjugation on siRNA backbone have been reviewed elsewhere [9].

## 2. The siRNA Conjugation Strategy

### 2.1. Structural Diversity of the siRNA Duplex

**Figure 1:**
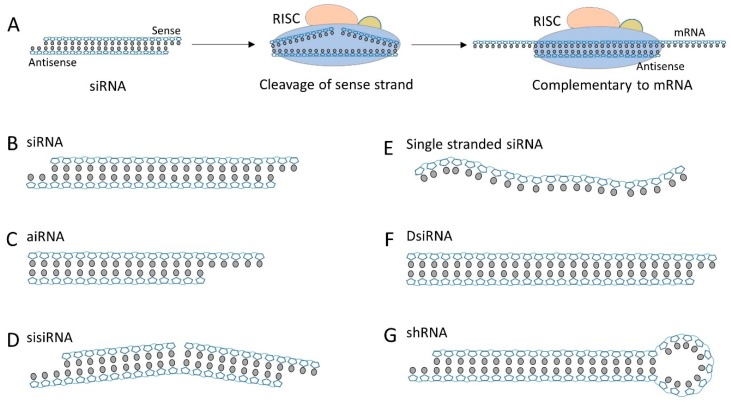
(**A**) A simplified schematic for the RNAi pathway. (**B**) The canonical siRNA that has 19 bp sequence plus a unilateral 2 nt overhang at 3′ end. (**C**) Asymmetric siRNA (aiRNA) with a 20 nt antisense strand and 15 nt sense strand. (**D**) Small internally segmented RNA (sisiRNA) containing the segmented sense strand. (**E**) The single-stranded siRNA that can engaging RNAi machinery. (**F**) Dicer substrate siRNA (DsiRNA) can be processed into mature canonical siRNA by Dicer cleavage. (**G**) Short hairpin RNA (shRNA) has a siRNA duplex plus a hairpin loop.

The endogenous RNAi pathway is a long biological process that involves sequential processing of long dsRNA into short 19-21-base pair (bp) siRNA, associating with argonaute-containing RNA-induced silencing complex (RISC), degrading sense strand thereby liberating the single-stranded antisense, and the cleavage of mRNA transcript by the activated RISC through sequence complementarity mechanism (Figure 1A) [10]. The stepwise process leads to the development of a diverse structures of siRNA duplexes that can step into RNAi pathway at different stages. The canonic siRNA has a structure of 19–21 nt duplex plus 2-nt 3′ overhang (Figure 1B). Its structure is same to that of dicer processed short dsRNA and can be directly integrated into RISC for gene silencing. It is so far the most widely used siRNA scaffold due to its feasibility of synthesis and modification. However, some limitations of canonic siRNA, especially the off-target effect, has been acknowledged in recent publications [11,12]. Other siRNA scaffolds have been developed to circumvent the limitations and hopefully can drive siRNA closer to the clinical application. Asymmetric interfering RNA (aiRNA) possesses as short sense strand (15–16 nt) and regular length antisense strand (20–22 nt) (Figure 1C) [13]. The asymmetric structure contains an atypical long overhang, improving antisense selection and reducing the off-target effect of gene silencing. Chu et al. demonstrated that aiRNA was readily accommodated by RISC and exhibited a similar gene silencing potency to the canonical siRNA [14]. With the same concept, Bramsen et al. hypothesized that instead of shortening the sense strand, the undesired activity of sense strand (off-target effect) could be reduced by segmenting the 21 nt sense sequence into two smaller sequences [15]. The small internally segmented interfering RNA (sisiRNA) designed by Bramsen et al. is a highly potent siRNA alternative that is capable of knockdown of the endogenous gene expression to the level of canonical siRNA, while abolishing the off-target silencing effect induced by complementary senses strand (Figure 1D). Because of the abrogation of sense strand activity, aiRNA and sisiRNA have been considered as useful alternative of canonical siRNA and been widely used for conjugation. A final approach for reducing off-target effect of siRNA is to completely abolish the sense strand and use the antisense strand as a single-strand siRNA (Figure 1E). With appropriate chemical modification and structural optimization, the single-strand siRNA can silence the gene expression in animals in the absence of cationic lipid [16].

Dicer substrate siRNA (DsiRNA) is a short 27 nt dsRNA duplex that has more potent gene silencing capability than canonical 21 nt siRNA (Figure 1F) [17]. DsiRNA is a substrate of Dicer. It is processed by Dicer to generate a canonical 21 nt siRNA and subsequently pass over to RSCI for RNAi. It is believed that the tandem biological process and a potential secondary role of Dicer is responsible for the enhanced gene silencing effect (up to 100-fold more potent) compared to the canonical siRNA (which engages in the RNAi pathway without Dicer participation) [18]. Short hairpin RNA (shRNA) is another siRNA scaffold that need to be processed by Dicer before engaging in RNAi pathway (Figure 1G). shRNA is a 19 nt duplex plus a tight hairpin turn. This duplex is generally annealed from a long single-strand RNA [19]. Similar to DsiRNA, shRNA has to be processed by Dicer and convert to canonical 21 nt siRNA for target-gene silencing. Despite the existence of similar Dicer-processing procedures to DsiRNA, shRNA does not show superior potency compared to canonical siRNA, which might be due to the loop structure and its weaker affinity to Dicer enzyme [20]. Expression of shRNA in cells is typically accomplished by delivery of plasmids encoding shRNA gene, but shRNA is also often exogenously synthesized for modification and delivery. 

### 2.2. Conjugation Sites and Linkers

**Figure 2:**
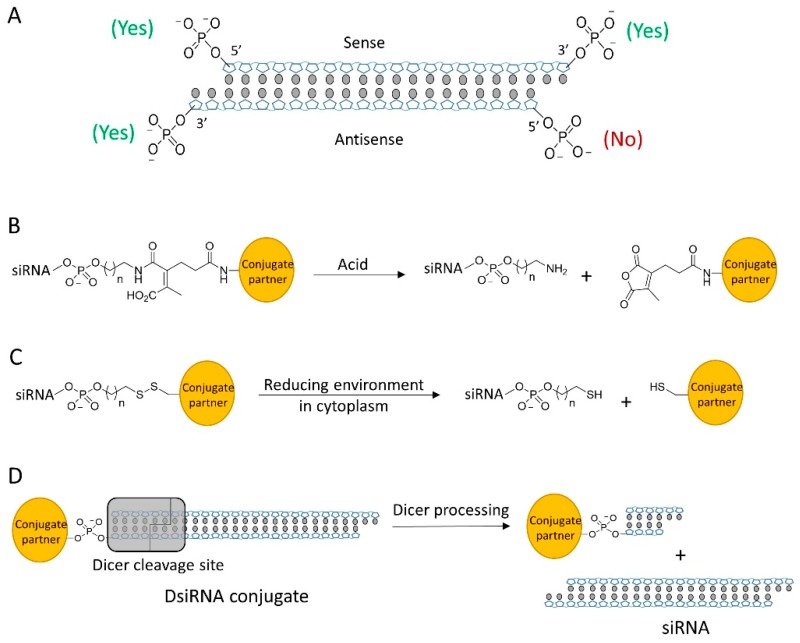
(**A**) siRNA contains 4 terminal phosphate groups for modification, but only 3 sites are tolerable for bioconjugation. (**B**) Acid-sensitive linker cleaved by acid. (**C**) Disulfide linker can be cleaved in cytosol to release siRNA. (**D**) DsiRNA conjugate is processed by Dicer to release mature siRNA.

siRNA is the duplex of two complementary strands of RNAs (sense and antisense strand). Structurally, siRNA has four terminal phosphate groups available for conjugation (Figure 2A). Previous studies have demonstrated that the 5′ end of the antisense strand is essential for the initiation of RNAi machinery and will not tolerate any chemical modification or conjugation [21,22,23]. The 5′ and 3′ ends of the sense strand and the 3′ end of the antisense strand have been considered as potential sites for conjugation. Due to the central role of the antisense strand in RNAi machinery, conjugation on 3′ end of antisense strand was rarely reported. Scientist generally conjugated siRNA at either the 3′ or 5″ end of sense strand to minimize the modification effect to the silencing potency. It is worthy notice that conjugation sites can also be located on the backbone of siRNA such as the 2′-OH of ribose, phosphate groups and the bases [24]. The chemical groups on backbone provide better flexibility to the selection of conjugate position and site number, having potential as a major strategy of siRNA conjugation in future.

Conjugation strategies of the siRNA bioconjugates in literatures are summarized in Table 1. Most of the siRNA bioconjugate employ cleavable linkage that enables the bioconjugate to shake off the conjugation partner for efficient accommodation into RISC. The common cleavable linkage includes acid labile linkers and disulfide linkages. The acid labile linkage is expected to be cleaved in the acidic endosomal/lysosomal compartments (Figure 2B) whereas the disulfide linker would be cleaved at the reductive environment of cytosolic space (Figure 2C). Some siRNA bioconjugate can be processed by Dicer and generate a mature siRNA (Figure 2D) [25]. This type of siRNA bioconjugates generally either has a longer siRNA scaffold such as DsiRNA or possesses a special linkage design to allow Dicer recognition [26,27]. It was reported that chemical conjugation on siRNA terminus could also enhance the nuclease resistance and increase the stability of siRNA in biological fluids [28]. However, it should be noted that chemical modifications of siRNA backbone are still desirable for fully protecting siRNA from degradation, especially against the endonuclease [29]. 

## 3. siRNA Conjugated with Ligand for Targeted Delivery

### 3.1. siRNA Conjugated with Immunoprotein

**Figure 3:**
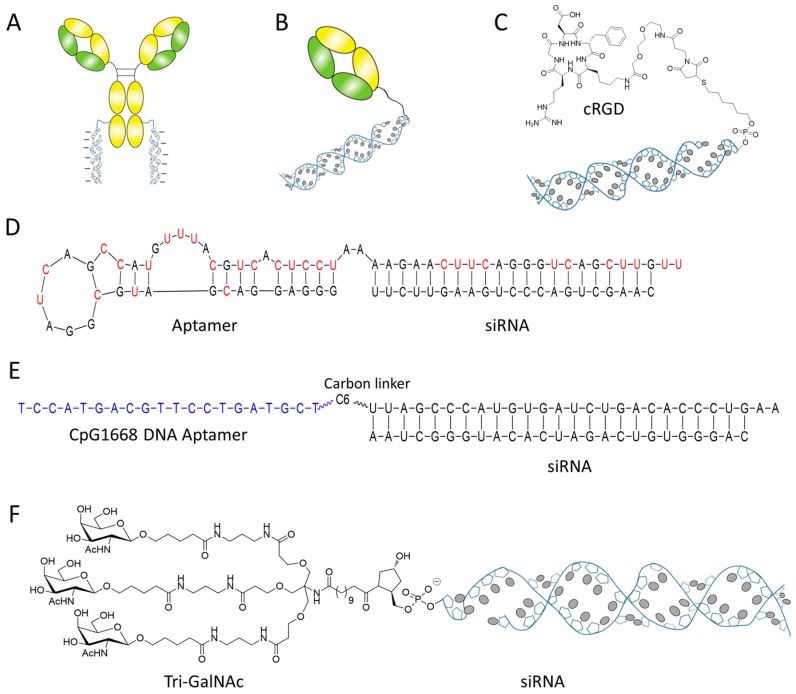
siRNA conjugated with ligands. (**A**) siRNA is directly conjugated with antibody via cleavable or noncleavable linker. (**B**) siRNA conjugated with immunoprotein Fab. (**C**) A targeting peptide ligand cRGD was conjugated with siRNA by noncleavable thioester bond. (**D**) The sequence and structure of a PSMA targeting aptamer siRNA chimera. (**E**) siRNA is conjugated with a DNA aptamer by carbon linker. (**F**) The structure of tri-GalNAc siRNA conjugate.

Given the high degree of specificity and affinity of antibody to its antigen, antibody has been established as the gold standard targeting ligand in drug delivery. Several groups have already developed the antibody based siRNA delivery technology by conjugating antibody with cationic materials for siRNA complexing and delivery [30,31,32]. Despite representing an intriguing proof-of-concept study, this method relies on nonspecific electrostatic interactions between cationic materials and siRNA, which may result in heterogeneous aggregates and eventually affects the clearance, toxicity and efficacy of the bioconjugate [33]. Inspired by the clinical success of antibody–drug conjugate, scientists from Genentech, Inc. relies on their THIOMAB technology that used in clinic for antibody–drug conjugate, covalently conjugated the chemically stabilized siRNA to the discrete position of antibody, eventually developed a homogenously pure antibody siRNA conjugate (ARC) with defined antibody/siRNA stoichiometry (Figure 3A) [34]. The study revealed that both antibody and siRNA in ARC maintained normal function in the conjugate. Covalently coupling of siRNA to an antibody, either by cleavable or noncleavable linker, did not impair the ability of the delivered siRNAs to mediate RNAi. The broad assessment of this technology on seven targets revealed that ARC targeting some receptors (TENB2 and NaPi2b) mediated moderate silencing efficacy, but the optimal silencing seems be restricted by the poor ARC egression from endosome [34].

Takeda Pharmaceutical Company has developed an anti-CD71 Fab siRNA bioconjugate for the delivery of siRNA to cardiac and skeletal muscles (Figure 3B) [35]. Similar to the above ARC, this siRNA bioconjugate only showed the silencing activities of maximally 40% on mouse primary cells, probably due to the limiting factor of endosomal escape. Encouragingly, this siRNA bioconjugate showed durable gene silencing in the heart and skeletal muscle for one month after intravenous administration, which indicated the advantage and usefulness of the bioconjugate in gene specific silencing and disease treatment, although the challenge remains [35].

### 3.2. siRNA Conjugated with Peptide Ligand

Compared with immunoproteins, peptides have low molecular weight, are compact in size, cheap, and easy to synthesize. With the widespread application of phase display technology, large numbers of peptide ligands have been identified to target a broad spectra of receptors and proteins [36]. Despite simple in structure, peptide ligands, such as BBN [7,8,9,10,11,12,13,14], can exhibit the high specificity and affinity to antigens comparable to the antibodies [37]. Peptide has become one of the most common ligands used for drug conjugate and delivery [38,39].

Cersarone et al. conjugated siRNA with an insulin-like growth factor 1 (IGF1) mimetic peptide that can recognize and bind with IGF1 receptor (IGF1R) [40]. The peptide siRNA conjugate was showed to knock down the target gene by 35 to 45% in IGF1R positive MCF-7 cells without the assistance of any transfection reagent. In another example, an integrin receptor-targeting peptide cRGD has been conjugated to the sense strand of anti-VEGFR2 siRNA and applied to integrin positive human umbilical vein endothelial cells (HUVEC) (Figure 3C) [41]. The cRGD-siRNA conjugate can selectively bind with HUVEC cells and down-regulate the VEGF gene by 40 to 55% without using any transfection agent. Impressively, this siRNA bioconjugate also showed a profound anti-tumor efficacy in vivo and reduced the overall tumor volume by 70 to 90% [41]. The therapeutic effect of cRGD siRNA conjugate can be further enhanced by engineering multivalent cRGD that has higher affinity and targetability to the receptor [42]. Alam et al. constructed the multivalent cRGD siRNA conjugate and compared the gene silencing effect with the monovalent bioconjugate [43]. Although all siRNA bioconjugates were taken up by M21+ cells, there were notable differences in their RNAi effectiveness. The tri- and tetravalent cRGD siRNA conjugates produced progressive, dose-dependent silencing activity, while the mono- and bivalent version had little effect. It is notify that the peptide siRNA conjugates were taken up by a caveolar endocytotic pathway and primarily entrapped in cytosolic compartment [43]. Gandioso et al. conjugated siRNAs to three well-known peptide ligands—cRGD, octreotide, and Tat-AHNP—and investigated the impact of endosomal entrapment to silencing effect [44]. It was found that all the three peptide siRNA conjugates showed weak or moderate silencing effect even at concentration of 1 µM. In contrast, the mRNA level can be suppressed to less than 5% if siRNA bioconjugate was applied in the presence of transfection agents (for endosomal escape). Just like the antibody siRNA conjugate, the poor egression from endosome remains the limiting factor to restrict the therapeutic potential of peptide siRNA conjugate.

Many groups start to investigate new approaches to enhance the intracellular delivery efficiency of peptide siRNA conjugate by either conjugating or complexing peptide siRNA with cationic materials. Nakamoto et al. tethered siRNA with both cRGD ligand and oligospermine (a cationic short-chain polymer for endosomal escape). They claimed that the new construct can overcome the low membrane permeability of siRNA (better endosomal escape) and showed sufficient gene silencing efficiency at final concentration of 250 nM in the absence of any transfection agent [45]. Similarly, peptide siRNA conjugates such as LHRH-siRNA or cRGD-siRNA have been complexed with cationic polymer PEI or lipid to form the targeting nanoparticle for better endosomal release and delivery [46,47].

### 3.3. siRNA-Aptamer Bioconjugate

Aptamer is a big class of RNA or DNA oligonucleotides that have high selective affinity to the target proteins or receptors. As the nucleic acid versions of antibodies, aptamer possesses the unique characteristics, such as easy synthesis, lack of immunogenicity, and small size, which makes it more adaptable for drug conjugation and delivery [48]. Actually, a multitude of aptamers have been utilized for delivery of a variety of agents including siRNA. Compared to antibody or proteins, chemical conjugation or siRNA tethering to aptamer is very simple and straight forward. siRNA was attached to the aptamer either through direct conjugation to the end of aptamer chain or through its complementary assembly to append with the aptamer. The first siRNA aptamer conjugate was constructed using the latter strategy in which McNamara et al. directly in vitro transcripted prostate-specific membrane antigen (PSMA) aptamer and sense strand of siRNA as a single chain, and then annealed with antisense strand of siRNA to form an aptamer siRNA chimera-targeting prostate PSMA (Figure 3D) [25]. Several research groups have followed the same methods and constructed distinct aptamer siRNA chimeras for delivery siRNA into tumor cells [49,50,51,52]. In some cases, when DNA aptamer was used as a targeting ligand, chemical linker has to be utilized to link siRNA and aptamer together [53,54]. Yu group has conjugated a DNA aptamer CpG1668 with anti-Stat3 siRNA by a carbon linker (six C3 units) (Figure 3E) [53]. The flexibility of carbon linker preserved the immunostimulatory properties of CpG1668 and at same while allow Dicer processing, synergistically enhancing antitumor immune responses. Functional optimization of siRNA aptamer conjugate has also been carried out in some groups [55]. For example, Giangrande’s group truncated the PSMA siRNA chimera to a smaller and more compact chimera without impairing the binding affinity [56]. Gmeiner’s group dimerized two aptamer on single construct to enhance the chimera’s targetability [57]. Gilboa’s group annealed two copies of siRNA with one aptamer in order to increase the drug loading efficiency [55]. Some other minor optimizations such as linker design and new conjugation chemistry, have also been reported [58,59]. 

### 3.4. Small Molecules as Ligand for siRNA Conjugation

Despite light molecular weight, small molecules can be a highly specific and strong ligand for siRNA delivery. Low group conjugated the sense strand of siRNA with small molecular ligand folic acid and DUPA, respectively [60]. The siRNA has been efficiently targeted to the malignant cells that overexpress the corresponding receptors (folic acid receptor and PSMA receptor), but the siRNA was predominately entrapped in the acidic lysosome compartment. Similarly, Dohen added a PEG linker between folic acid and siRNA [61]. Synthesized with the precision of solid phase chemistry, the new bioconjugate represented a structural defined, monodisperse folate–PEG–siRNA conjugate that showed a specific binding and internalization into folic acid receptor expressing cells in vitro. However, the uptake by endocytosis did not result in significant gene silencing. This bioconjugate was found trapped in the lysosomal vesicles, consistent with the observation by Low’s group [60]. The lack of endosomal escape functionality did not confer on siRNA the access to cytosol where the RISC is located and the RNAi events take place. Combination with agents enabling efficient endosomal escape seems always necessary for siRNA bioconjugate to elicit its therapeutic effect [61,62,63].

However, some hepatocyte targeting carbohydrate siRNA conjugates are the exception. Many groups have demonstrated the efficient intracellular delivery of siRNA by directly conjugating siRNA to galactose (M6P) or galactose derivatives, the ligand of the asialoglycoprotein receptor (ASGPR) [64,65,66,67,68]. Initially, monovalent galactose was conjugated with siRNA for delivery but only moderate knockdown effect was observed (25% of inhibition) [65,66]. Nair et al. introduced the multivalent GalNAc (*N*-acetylgalactosamine), a derivative of galactose showing higher binding affinity to ASGPR [69,70], to the 3′ end of sense strand of siRNA via a noncleavable trans-4-hydroxyprolinol (tHP) linker (Figure 3F) [64]. Single dosing of multivalent GalNAc siRNA conjugate showed robust liver-specific uptake and specific mRNA suppression in mice. Chronic weekly dosing resulted in sustained gene silencing for over nine months without adverse effects in rodents. This siRNA bioconjugate has already showed the positive initial phase 2 data, and some other bioconjugates based on the same platform targeting other disease genes have also entered phase 1 clinical trials for the treatment of hemophilia and hypercholesterolemia [71].

## 4. Lipid–siRNA Conjugate 

### 4.1. Cholesterol siRNA Conjugate

siRNA is a highly hydrophilic, polyanionic macromolecule that possesses poor pharmacological properties. Unmodified siRNA generally is reluctant to bind with any serum protein, thus being quickly excreted from blood circulation by renal filtration. Without chemical modification or carrier assistance, siRNA shows a plasma half-life as short as 5–10 min [72]. In contrary, small molecular drugs avidly bind with serum proteins and circulate in blood with a half-life ranging from several hours to days, although it has a much smaller molecular weight than siRNA [73]. It rises the necessity of chemical conjugation with lipid to increase siRNA lipophilicity and confer on siRNA the ‘drug-like’ property for efficient delivery. 

**Figure 4:**
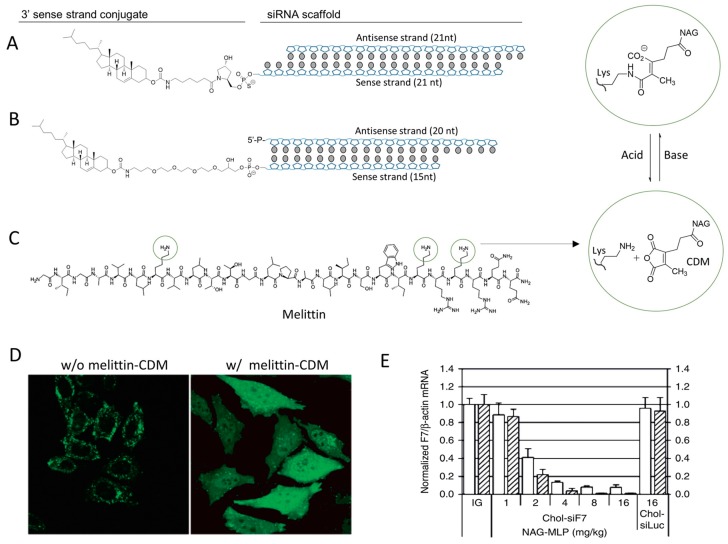
(**A**) The chemical structure of cholesterol siRNA conjugate. (**B**) The structure of cholesterol conjugated with aiRNA. (**C**) The chemical structure of pore forming peptide melittin and the mechanism of pH-sensitive CDM chemistry. The three potential modification sites of melittin are cyclized. (**D**) Melittin–CDM derivative enhances the escape efficacy of cargo from endosome/lysosome. FITC labeled cargo shows a punctuated distribution in cells (left), but localizes to the whole cell in the assistance of melittin–CDM derivative. (**E**) NAG-MLP (melittin–CDM derivative, dosing from 1 to 16 mg/kg) dramatically improves the in vivo gene silencing effect of chol-siRNA (chol-siF7). Adapted with permission from [86,87].

Scientists at Alnylam Pharmaceutical Inc. conjugated cholesterol to the 3′ end of sense strand of siRNA by a short trans-4-hydroxylprolinol linker (Figure 4A) [74,75]. This lipophilic siRNA (chol-siRNA) can avidly bind with serum protein and exhibit a plasma half-life of 90 minutes [74]. It shows an enhanced accumulation in liver and, subsequently, gene silencing in vivo. The efficient delivery of the chol-siRNA was highly dependent on the presence of the cholesterol moiety. The cholesterol incorporates into lipoprotein and forms a lipoprotein/cholesterol particle, which hijack the natural lipid transport pathway and deliver siRNA into liver by the lipoprotein receptor–mediated endocytosis [74]. It was reported that chol-siRNA could bind with different types of lipoproteins including HDL (high-density lipoprotein) and LDL (low-density lipoprotein). Both forms can mediate differential uptake pathway in vivo. For example, LDL particles containing chol-siRNA predominately deposit in the liver because of the high expression of LDL receptor in liver cells. On the other hand, HDL particle mediates the delivery of chol-siRNA to many other organs including adrenal, ovary, kidney, and intestine. The uptake of the HDL particle is mainly mediated by the scavenger receptor SR-BI (scavenger receptor class B type I). It was evidenced that the biodistribution profiles of chol-siRNA in vivo correlated well with the expression of the SR-BI receptor in tissues [74]. Besides lipoprotein, chol-siRNA also strongly binds to serum protein albumin, with an estimated dissociation constant (K_d_) of 1 µM [72]. Albumin is the most abundant endogenous serum protein and has a circulation half-life of ~20 days [76]. The bound of chol-siRNA to albumin would extensively elongate the serum half-life, which is responsible for broadening the tissue biodistribution beyond the liver. The heterogeneity of particle formation in serum raises a necessity of preparing a ‘pure form’ that can take advantage the most efficient uptake pathway for siRNA delivery [77,78,79]. Lipoprotein nanoparticles made from ex vivo-assembly of chol-siRNA and exogenous LDL (or HDL) have been developed and evaluated [77,78]. These ex vivo-assembled lipoprotein/chol-siRNA typically has a more uniform particle size and shows better delivery efficacy than the chol-siRNA alone, especially when the targeted delivery organ is the hard reachable tissues such as brain. It might be due to the homogenous structure and better colloidal stability of the ex vivo-assembled lipoprotein particle. 

Khvorova’s group conjugated cholesterol with aiRNA and generated a new chol-siRNA scaffold, annotated hsiRNA, that can be efficiently uptake into cell in vitro and in vivo without aid of any transfection agents (Figure 4B) [80,81,82,83,84]. Compared to the canonical chol-siRNA, hsiRNA has fewer overall negative charges on siRNA, while maintaining RNAi potency. Moreover, the asymmetric structure of hsiRNA contains a 5 nt single-strand tail which can be phosphorothioatedly modified and enhanced the cell internalization of siRNA following a mechanism similar to that of the fully phosphorothiolated antisense oligonucleotide [85]. Because of the superior structural design, hsiRNA can bind and internalize into cell in minutes after exposure to cells [80]. 

The therapeutic efficacy of chol-siRNA can be further increased by adding endosome-escape enhancer to help chol-siRNA escape from endosome/lysosome compartments. Arrowhead Inc. developed a natural pore-forming peptide melittin (bee venom) derivative to co-administrate with chol-siRNA [86]. The derivative is a CDM (carboxylated dimethyl maleic acid, pH-sensitive group) modified melittin that can be deprotected and reversed into natural melittin in the acidic environment of the endosome, thus activating its pore forming activity and lysis the endosome vesicles (Figure 4C,D) [87]. The simple co-injection of this melittin derivative (NAG-MLP) with chol-siRNA has increased the gene silencing efficacy by ~25-fold compared to the injection of chol-siRNA alone (Figure 4E), without toxicity in mice and nonhuman primates [86]. This combination has been developed as a RNAi therapeutic for the treatment of chronic hepatitis B virus (HBV) infection and promoted into clinic trail under the name of ARC-520. In the phase I study consisting of 54 healthy volunteers, single injection of ARC-520 was well tolerated within an intravenous dose of 0.01–4.0 mg/kg [7]. The adverse-event frequency was same as placebo and no serious adverse events were observed. A phase 2a clinic trial revealed that ARC-520 can reduce the expression of s-antigen, e-antigen, and correlated antigen in human after a single dose injection, a positive evidence correlating to the pharmacological outcome of siRNA mediated HBV protein suppression [6]. Unfortunately, the clinical trial was hold by FDA in 2017, likely due to the concerns of immunogenicity and toxicity invited by this exogenous toxin. 

### 4.2. Bioconjugation with Other Lipids

**Figure 5:**
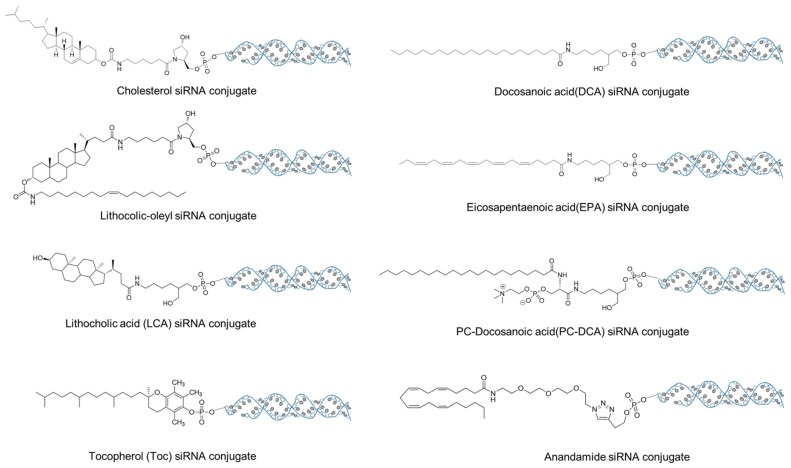
The chemical structures of the lipids utilized for siRNA bioconjugate.

The chemical structure of lipid has a profound effect on the efficacy of siRNA in vivo delivery. It has been reported that the intravenously injected chol-siRNA exist as three forms (complex with LDL, HDL, and albumin), all of which are correlated with different transport pathways and biodistribution profiles [74]. The interaction between the lipids and proteins (LDL, HDL, and albumin) is driven by the lipophilicity of lipid and highly dependent on the lipidoid structure [88,89]. It has been demonstrated that siRNA conjugated with cholesterol, lithocolic-oleyl (Figure 4), and all other highly lipophilic lipids prefers binding with LDL, and thus go to the LDL-mediated lipid transport pathway and predominately deposit in the liver [74]. Lipid with moderate hydrophobicity such as lithocholic acid (LCA, Figure 5) has better affinity to HDL and albumin. Its complex hijacks a different transport pathways and eventually deposits at a widespread tissues including liver, kidney, ovary, intestine, and others [88]. 

Besides conferring the lipophilicity, lipids also provide siRNA the targeting capability to the organs and cells. For example, siRNA conjugated with α-tocopherol (vitamin E) can bind with α-tocopherol-associated proteins in serum and take advantage of its pathway for hepatic uptake and liver delivery [90]. Similarly, DCA-siRNA (Figure 5) preferentially accumulated in heart, whereas EPA-siRNA mainly deposited in lung [89]. Even the same lipid, once slightly modified, can target to a different organs or tissues. PC-DCA-siRNA, which adds a phosphocholine (PC) to DCA structure (Figure 5), showed a different biodistribution profile to DC-siRNA and preferentially deposited in muscle, fat and adrenal gland [89,91]. The selectivity of the lipid to a specific tissue or cell type might be due to the affinity of the lipid structure to a membrane of specific cell type. This type of affinity between lipid and membrane has been utilized by Willibald et al. to targetedly deliver anandamide-siRNA into neuronal and immune cells [92].

## 5. siRNA Conjugated with Cell-Penetrating Peptides

### 5.1. Conjugate with Cationic CPP

**Figure 6:**
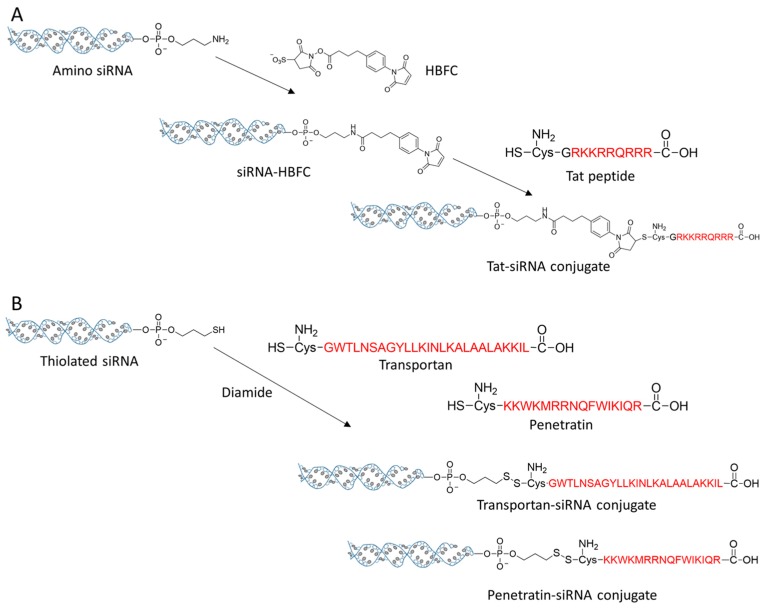
(**A**) The conjugation strategy of Tat-siRNA. (**B**) Transportan and penetratin were conjugated with siRNA by the disulfide linkers.

CPP is a class of cationic peptides that can penetrate cell membrane and transport cargo into cell cytosol. The well-known Tat peptide (sequence: RKKRRQRRR) was discovered from Tat protein of human immunodeficiency virus type-1 (HIV-1) in the late 1990s [93]. Penetratin is a CPP originated from the homeodomain protein of Antennapaedia, and another famous CPP, transportan, is a hybrid of amino acid sequence from glanin and mastroparan [94,95]. All these CPPs have been utilized for conjugation with siRNA for efficient delivery. Chiu et al. conjugated Tat peptide to the 3′- end of antisense strand of siRNA by a noncleavable linker HBFC (succinimidyl 4-(p-maleimidophenyl) butyrate) (Figure 6A) [96]. After incubation with cells, Tat-siRNA conjugate was quickly translocated into cells, and the extent of uptake was positively correlated with the dose of Tat-siRNA and the elapsed time of treatment. CPP conjugation with siRNA seems not seriously impairing the gene silencing potency of the siRNA. Tat-siRNA exhibited a potent and dose-dependent silencing effect to endogenous cyclin-dependent kinase 9 (CDK9) gene on HeLa cells. A cleavable, disulfide linkage on the 5′ end of sense strand is more popular for siRNA bioconjugation. This type of bioconjugate has been applied to conjugate siRNA with penetratin and transportan, respectively (Figure 6B) [97,98]. The new siRNA bioconjugate improved the intracellular delivery efficacy of siRNA by shaking off CPP and releasing intact siRNA in the reductive cytoplasmic environment. It has been reported that the gene silencing efficacy of siRNA bioconjugate by this strategy is comparable to that of the cationic lipid mediated transfection [98]. However, it worth notify that some CPP siRNA conjugate, such as penetratin–siRNA conjugate, might cause the elevated secretion of inflammatory cytokines, suggesting the activation of innate immune response [99]. 

### 5.2. Conjugate with Activatable CPP

Activatable CPP is a CPP prodrug in which the cationic charges are masked by counterions or protecting groups thus the whole peptide possesses weak or no cationic charges [100,101]. Once activated, the counterions or protecting groups are removed and the whole peptide transforms into the full functional cationic CPP, eventually carrying the siRNA cargo into cells. The first activatable CPP is a hairpin-structured peptide, consisting a polycationic CPP domain and polyanionic domain plus an enzyme cleavable peptide loop linkage [102]. Since the positive charges of the CPP domain is neutralized by the intramolecular polyanionic domain by electrostatic interaction, the activatable CPP and its cargo will not be taken up by cells, thus dramatically reduce the nonspecific uptake and corresponding toxicity. Li et al. added the matrix metalloproteinase-2 (MMP2) substrate sequence into the loop and utilized the activatable CPP to deliver hTERT siRNA into hepatocellular carcinoma cells (SMMC-7721) that overexpress MMP2 [103]. Studies from Roger Y. Tsien’s group revealed that the cell uptake of this bioconjugate increased > 10-fold when the linker was cleaved by MMP2 [104]. In vivo study showed that the tumor uptake of activatable CPP cargo bioconjugate is 3.1 fold higher than that of the control peptide with a scrambled, uncleavable linker loop, which demonstrated the importance of linker cleavage and CPP activation. 

Activatable CPP can also be made by reversibly masking the amino acid residues (especially Lys and Arg) of CPP by chemical protecting groups. Due to the chemical reluctance of Arg residue, most of the modification was made on ε-amino group of Lys [105]. However, polylysine is not an efficient CPP for siRNA delivery. siRNA delivery mediated by this type of activatable CPP is rarely reported.

## 6. siRNA Conjugated with Polymer

### 6.1. Polymer–siRNA Conjugate

**Figure 7:**
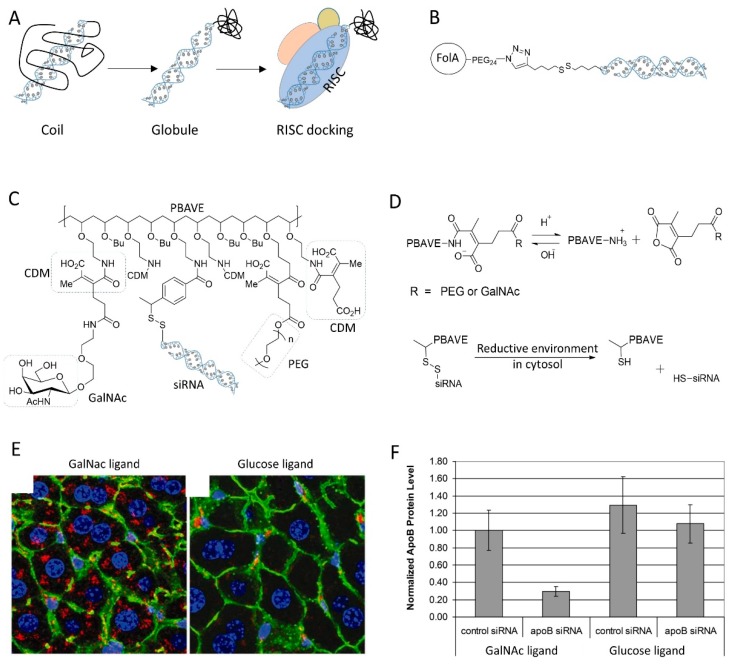
(**A**) Controlling gene silencing activity of siRNA by conjugation with thermoresponsive polymer that has coil–globule transition behavior. (**B**) The structure of folic acid PEG siRNA conjugate. (**C**) The chemical structure of dynamic polyconjugate (DPC) using PBAVE as backbone polymer. (**D**) Depicted is the decomposition of DPC inside cells, mainly by reactions of CDM under acid condition and disulfide under reductive environment in cytosol. (**E**) Targeted delivery of DPC to liver was mediated by the ligand GalNac. Glucose ligand was used as a control. (**F**) Knockdown of target gene expression in livers of mice after i.v. injection of DPC. Adapted with permission from [111].

Due to high hydrophilicity, siRNA suffers from the poor delivery efficiency and bioavailability. It is believed that the problem is caused by rapid renal filtration and fast elimination of siRNA from blood circulation. Conjugation with polymer, especially PEG, is a common concept to increase drug’s half-life in blood and enhance the pharmacokinetic profiles [106]. It has been reported that siRNA conjugated with 20K PEG has a much slower clearance rate in vivo (~50% remained at 1 h postinjection) than that of the uncoupled siRNA (<10% left at 15 min) [107]. In vivo fluorescent imaging of the living animals also revealed that PEG20K siRNA conjugate showed a broaden distribution in peripheral tissues, liver, kidney, spleen, and lung, evidence of the improved siRNA bioavailability and delivery efficacy. Although better bioavailability, PEGylation of siRNA generally attenuates the gene silencing potency of siRNA, especially when conjugated in a noncleavable manner, since the steric clash of PEG prevents siRNA accommodation into RISC. Most of the well-defined PEG siRNA conjugate employ a disulfide linker to control the integrity of bioconjugate during circulation and siRNA release inside cells [108]. Nishiyama group developed a strategy to use the steric hindrance of polymer to control the bioactivity of the vicinal siRNA molecule (Figure 7A). Instead of PEG polymer, they utilized a thermoresponsive polymer PNIPAAm to conjugate with siRNA [109]. At temperature (T) less than the lower critical solution temperature (LCST), PNIPAAm exhibits as a random coil which has a large body and prevent siRNA docking into RISC. At T > LCST, the polymer chain stack together to form a globule. It has a compact size and allows a ready access of siRNA into gene silencing machinery (RISC). The coil–globule transition has successfully controlled the gene silencing efficacy of the siRNA bioconjugate with response of temperature. 

To better enhance the bioavailability and targetability, the polymer–siRNA conjugate has been grafted with ligand for targeting specific tissues or organs. Dohmen et al. covalently tethered the folic acid to the PEG2K-siRNA conjugate by click chemistry, yielding a monodisperse ligand–PEG–siRNA conjugate (Figure 7B) [61]. This ligand–PEG–siRNA conjugates showed highly selectivity to folate receptor positive KB cells, whereas the normal PEG siRNA conjugate didn’t. However, the siRNA bioconjugate failed to knock down the target gene in KB cells, unless with assistance of cationic transfection reagent, probably due to the failure of endosomal escape. Instead, York et al. replaced the PEG polymer with HPMA-s-APMA copolymer [110]. Compared to PEG, the copolymer not only provides conjugate sites for folic acid and siRNA grafting, but also possesses inherent endosomolytic ability originated from APMA block.

### 6.2. Dynamic Polyconjugate of siRNA

To overcome the barriers during delivery, delivery systems generally have to harness the potentials of several different components for a safe and effective delivery of siRNA to target cells. As for a polymer–siRNA bioconjugate system, these essential components may include the ligand for targeting, the endosomolytic component for endosomal escape, siRNA as cargo, PEG chain to elongate blood retention, and potentially a hydrophobic lipid for better interaction with cell membrane. To yield this multiconjugate system, scientists generally subsequently couples all these components step by step into a polymeric backbone that contains many conjugate sites (such as polylysine). The conjugate reaction on the backbone is dynamic and it is hard to precisely control the conjugate position or the conjugate extent, which therefore has been named ‘dynamic polyconjugate’. However, the bioconjugate molecule generally is quick, small in size, and can be purified, chemically defined, and well-characterized, thus showing less heterogeneity than the cationic polymer/siRNA complex. 

Rozema et al. developed the first dynamic polyconjugates (DPC) for targeted in vivo delivery of siRNA to liver (Figure 7C) [111]. The polyconjugate was constructed by firstly disulfide linking siRNA to the polymeric backbone PBAVE (which is featured by many primary amine groups), followed by grafting PEG and NAG (liver tropic ligand) onto it by the acidic sensitive linker CDM. The linkage by CDM masked the cationic charges of PBAVE, thus deactivated its endosomolytic capability. At the acidic endosomal environment, the low pH induced the release of CDM linker and the bioactivity of PBAVE was recovered for endosome disruption (Figure 7D). The DPC specifically delivered its siRNA cargo to hepatocytes in vivo and effectively knockdown endogenous apoB gene in mouse liver after simple, low-pressure i.v. injection (Figure 7E,F). A multitude of similar polyconjugates have been developed for targeted siRNA delivery. All of them followed the same design logic, but used different polymeric backbones, such as polylysine, PAsp(DET), and poly amino amine [112,113,114,115]. Despite the great potential of these polyconjugate strategy, the safety issues, especially the immunogenicity, might be a concern, because the multimolecular siRNA conjugate in this platform may stimulate immune response through the activation of toll-like receptor 3 and/or protein kinase R [112]. 

## 7. siRNA Conjugated with Nanoparticles

### 7.1. Spherical siRNA Nanoparticle

**Figure 8:**
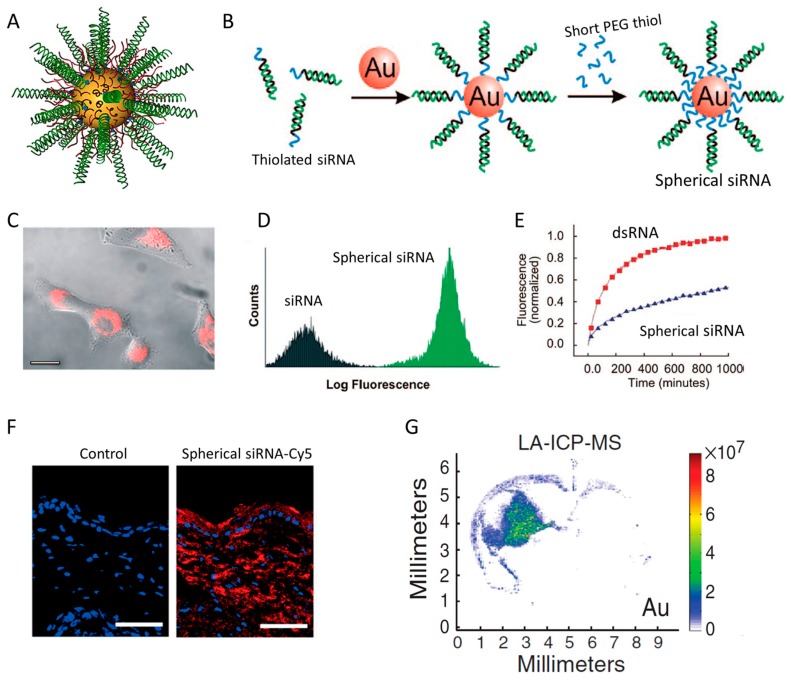
(**A**) The schematic structure of spherical siRNA nanoparticle. (**B**) Preparation of spherical siRNA by immobilizing siRNA and short-PEG to Au nanoparticle surface in two steps. (**C**,**D**) Spherical siRNA (Cy3-labeled) showed strong cell uptake on HeLa cells. (**E**) The structure of spherical siRNA (RNA–Au NPs) can protect siRNA (dsRNA) from degradation in serum. (**F**) Spherical siRNA penetrates through mouse skin and delivers siRNA into in the cytoplasm of epidermal cells. (**G**) The localization of Au content in coronal brain sections of mice injected intracranially with spherical siRNA. Adapted with permission from [117,119,121,126].

The linear nucleic acid such as siRNA typically requires the use of targeting ligand, lipid, or cationic polymers to overcome the Columbus repulsive forces between siRNA and the negatively charged cell membrane. Mirkin’s group discovered that the spherical nucleic acid (SNA) architecture (Figure 8A), in which siRNAs (or short DNA) are vertically assembled on Au nanoparticle in high density, overcome the repulsive force and can be delivered into cells with high efficiency (Figure 8B,C) [116,117,118]. The unique ensemble properties of the siRNA conjugate confers several important advantages in the context of drug delivery [119,120]. First, the 3D-based siRNA construct appears to be recognized by cells through a mechanism that is different from neither the receptor–mediated endocytosis by ligands nor the electrostatic interaction exploited by cationic materials. Study from Mirkin’s group revealed that the rapid cellular uptake and intracellular transport of SNA are mediated by the endocytosis and lipid raft correlated with Class A Scavenger Receptors (SR-A and CAV-1) [121]. Second, the high-density of identical siRNA molecules on spherical gold nanoparticle, typically 100 oligonucleotides per particle of 10 nm diameter [122], enables siRNAs to be delivered to the cell as a package, rather than the linear, single oligonucleotide that be recognized by cells one by one, which represents a more efficient delivery mode (Figure 8D) [121]. Indeed, within the same condition (PEI transfection), the spherical siRNA architecture underwent enhanced and more rapid uptake than the randomly assembled PEI–siRNA polyplexes that lack controlled architecture [123]. Thirdly, the crowd architecture also provides a steric hindrance that protect siRNA from the nuclease degradation (Figure 8E), which overcomes one of the most challenging problem associated with siRNA delivery [119]. With the aid of these unique properties, spherical siRNA nanoparticles overcome some of the most difficult delivery obstacles, such as skin barriers (Figure 8F) and the blood–brain barrier (Figure 8G), to knock-down endogenous disease-related genes for the treatment of psoriasis and glioblastoma [124,125,126]. 

It is worth noting that the delivery advantage of SNA is dominated by the 3D ensemble structure, rather than the Au nanoparticle core. By etching the Au core with KCN, Choi et al. created a “hollow SNA” and found that the hollow SNA can be uptake by cells with a rate and extent similar to the regular SNA [121]. Similar observation was also reported by other groups who constructed hollow silica siRNA spheres by dissolving the core of silica SNA with HF [127,128]. Moreover, neither the spherical shape or core materials is an important factor to govern the endocytosis. Advanced endocytic uptake has been reported on the SNA that siRNA are assembled on gold nanorod, liposome and globular protein [129,130,131].

### 7.2. siRNA Conjugated with Other Nanoparticles

In the past decades, many nanoparticles, including various polymeric nanoparticles, dendrimers, and inorganic nanoparticles, have been utilized for siRNA delivery. In most cases, siRNA was noncovalently complexed with nanoparticle, rather than immobilizing on the particle surface, likely due to the simplicity and effectiveness of this noncovalent approach. Nevertheless, siRNA nanoparticle conjugate has been developed in some groups for dual therapeutic and diagnostic analysis purposes. 

**Figure 9:**
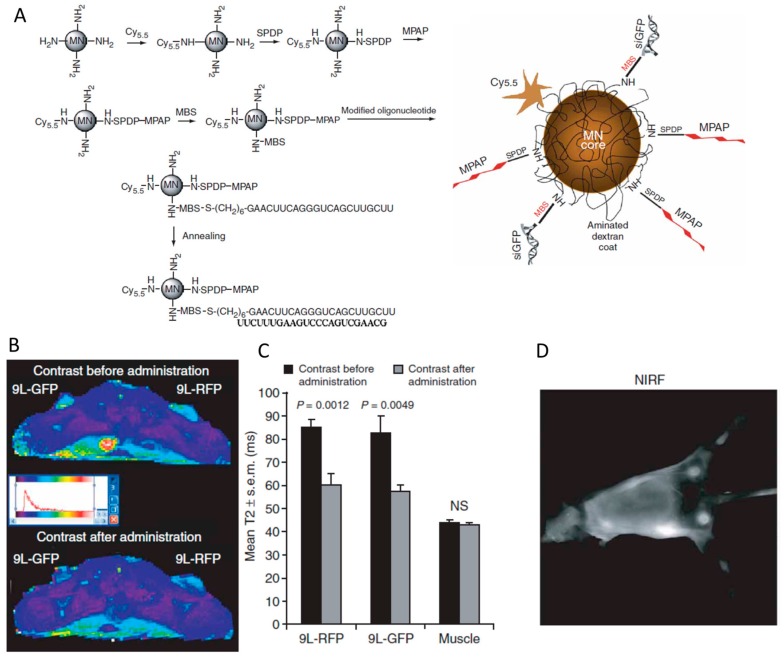
(**A**) The synthesis of the magnetic nanoparticle (MN) by decorating Cy5.5, MPAP, peptide and siRNA step by step. (**B**) Magnetic resonance imaging of mice bearing bilateral 9L-GFP and 9L-RFP tumors before and 24 h after the administration of MN-siRNA conjugate. (**C**) The tumors exhibit a significant drop in T2 relaxivity, whereas the muscle tissue remained unchanged. (**D**) In vivo NIR imaging of the tumors in mice. Adapted with permission from [132].

Magnetic nanoparticles (MN) have been intensively utilized for biomedical diagnosis in vivo by the method of magnetic resonance imaging (MRI). For visualization of siRNA biodistribution in mice, siRNA was conjugated with the iron oxide nanoparticle and intravenously injected into mice for imaging [132]. For siRNA immobilization, MN was functionalized with primary amine and activated with a heterofunctional cross-linker (MBS), which was then reacted with the thiol group of the antisense strand of siRNA to form a noncleavable thioester linkage (Figure 9A). Moreover, near-infrared dye Cy5.5 and membrane translocation peptide MPAP were also conjugated on the nanoparticle surface for NIR imaging and endosomal escape of nanoparticle (Figure 9A). The visualization of tumor were correlated very well by simultaneously imaging with MRI and near-infrared (NIR), because of successful accumulation of the nanoparticle at the tumor tissue (Figure 9B–D). Despite the noncleavable linkage in this experiment, a surprising silencing effect (>80%) was observed in vivo [132]. It is important to note that the steric clash from the bulky nanoparticle could be a dominated factor to impair the silencing efficacy in some cases. Derus et al. conjugated siRNA to quantum dot (QD) via noncleavable SMCC linker [133]. The nanoparticle could be successfully delivered into HeLa cells but no gene silencing effect was observed even in the presence of cationic transfection liposome. However, a significant reduction in target gene expression was observed when the siRNA was conjugated to QD through a disulfide linker for cytosolic release of siRNA from nanoparticle. Similarly, the importance of cleavable linkage on gene silencing effect has been demonstrated in the examples of siRNA conjugated with nanotube and dextran-nanoparticle [134,135].

## 8. Future Perspectives

Since the discovery of RNAi phenomena in 1998, siRNA has become one of the most popular tools for gene function study and also been extensively exploited for therapeutic use. Development of a safe and efficient delivery system remains one of the biggest barriers to turn siRNA into clinically acceptable drug. siRNA bioconjugate has many favorable characteristics including small in size, well-defined chemistry, homogeneity, and ease of formulation, making them very attractive for meeting the rigorous demands of drug manufacturing and clinical trials. In this regard, scientists from academy and pharmaceutical companies have proposed and investigated a wide variety of siRNA bioconjugate, some of which have already demonstrated very promising results in clinical trials [71]. To maximize the delivery efficiency of the bioconjugate, many parameters such as siRNA stability, the conjugate site of siRNA, the cleavability of linker, the space of linkage, the functionality of molecules to be conjugated, should be carefully screened and optimized. The structurally optimal siRNA bioconjugate could not only promote cell uptake specificity and efficiency, but also improve their pharmacokinetic behavior and tissue bioavailability, under the premise of gene silencing activity.

On the other hand, the potential immune stimulation and off-target properties of siRNA should also been considered. It has been reported that chemical modification of siRNA backbone such as 2′-fluoro and 2′-*O*-methyl substitution can reduce the acute immune response to the synthetic siRNA [136]. However, the immune response to the siRNA of terminal conjugation remains unclear. Previous studies have already revealed that administration of penetratin–siRNA conjugate could induce the expression of IFN-α and TNF-α [99]. It is believed that the inducer of immune stimulation might come from the foreign materials tethering with siRNA [137]. Silencing of the unwanted gene (off-target) by siRNA bioconjugate can also occur. A significant fraction of false positive hit might come from the incorporation of siRNA sense strand to RISC and the induction of off-target silencing the gene sequentially complemented to the sense strand [11]. Direct conjugation to sense strand or use of asymmetric siRNA scaffold could enhance the biased incorporation of antisense strand into RISC, resulting in a reduction of off-target effects. However, off-target silencing can be also observed in transcripts with ~7 nt of sequence complementarity at the 5′ end of siRNA antisense strand [12]. Altogether, siRNA bioconjugate could be a doubled-edged sword, but the benefits outweigh the risk. It worth further investigation, especially an appropriate selection of the foreign materials and conjugation chemistry, to translate this strategy from bench to market. 


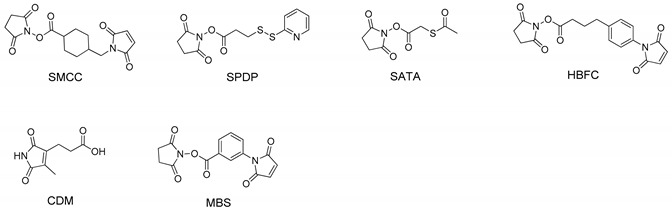


## Figures and Tables

**Table 1:** The linkage strategy of siRNA bioconjugates.

Strategy	siRNA Bioconjugate	Conjugate Site	Linker *	siRNA Scaffold	Reference
Ligand siRNA conjugate	Antibody-siRNA	3′ end of sense strand	SMCC (noncleavable) or SPDP (disulfide)	siRNA	[34]
	Fab-siRNA	3′ end of sense strand	DMSS (disulfide)	siRNA	[35]
	IGF1 mimetic peptide-siRNA	5′ end of sense strand	C6 carbon (noncleavable)	siRNA	[40]
	cRGD-siRNA	3′ end of sense strand	SMCC (noncleavable)	siRNA	[41,42,43]
	Octreotide-siRNA	5′ end of sense strand	Click linker (noncleavable)	siRNA	[44]
	Tat-AHNP-siRNA	5′ end of sense strand	Click linker (noncleavable)	siRNA	[44]
	cRGD-siRNA-spermine	5′ end of sense strand	Disulfide	siRNA	[45]
	LHRH peptide-siRNA	3′ end of sense strand	PEG-SPDP (disulfide)	siRNA	[46]
	Anti-PSMA aptamer-siRNA	5′ end of sense strand	RNA oligo (cleavable by Dicer)	siRNA	[25,56]
	CpG1668-siRNA	5′ end of sense strand	C6 carbon (noncleavable)	siRNA	[53]
	Folate-siRNA	5′ end of sense strand	PEG disulfide	siRNA	[60,61]
	DUPA-siRNA	5′ end of sense strand	Disulfide	siRNA	[61,62,63]
	M6P-siRNA	3′ end of sense strand	PEG disulfide	siRNA	[65,66]
	Lac-siRNA	5′ end of sense strand	β-thiopropionate (acid cleavable)	siRNA	[67]
	GalNac-siRNA	3′ end of sense strand	trans-4-hydroxyprolinol (noncleavable)	siRNA	[63,68,69]
Lipid siRNA conjugate	Chol-siRNA	3′ end of sense strand	trans-4-hydroxyprolinol (noncleavable)	siRNA	[72,74,75,78,79]
	hsiRNA	3′ end of sense strand	TEG (noncleavable)	aiRNA	[80,81,82,83,84]
	α-tocopherol-siRNA	5′ end of antisense strand	phosphodiester (noncleavable)	DsiRNA	[90]
	DCA, EPA or PC-DCA-siRNA	3′ end of sense strand	C7 linker (noncleavable)	aiRNA	[89,91]
	Anandamide-siRNA	3′ base of sense strand	Click linkage (noncleavable)	siRNA	[92]
CPP siRNA conjugate	Tat-siRNA	3′ end of antisense strand	HBFC (noncleavable)	siRNA	[96]
	Transportan-siRNA	5′ end of sense strand	Disulfide	siRNA	[97,98]
	Penetratin-siRNA	5′ end of sense strand	Disulfide	siRNA	[97,98]
Polymer siRNA conjugate	PEG-siRNA	3′ end of sense strand	C6 carbon (noncleavable)	siRNA	[107]
	PNIPAAm-siRNA	5′ end of sense strand	Click linker (noncleavable)	siRNA	[109]
	HPMA-s-APMA-siRNA	5′ end of sense strand	Disulfide	siRNA	[110]
	Dynamic polyconjugate	5′ end of sense strand	SATA (disulfide)	siRNA	[111,113,114,115]
	Releasable/enzyme-disrupting conjugate	n/a	CDM (acid cleavable)	siRNA	[112]
Nanoparticle siRNA conjugate	Spherical siRNA	3′ end of sense strand	Spacer 18 thiol-gold bond (noncleavable)	siRNA	[119]
	Magnetic nanoparticles-siRNA	5′ end of sense strand	MBS (noncleavable)	siRNA	[132]
	Quantum dot-siRNA	5′ end of sense strand	SMCC (noncleavable) or SPDP (cleavable)	siRNA	[133]
	Nanotube-siRNA	5′ end of sense strand	Disulfide	siRNA	[134]
	Dextran cage-siRNA	5′ end of sense and antisense strand	Photocleavable linker	siRNA	[135]

* The chemical structures of common linkers for siRNA bioconjugate.

## Abbreviations

nt	Nucleotide
FDA	Food and Drug Administration
RNAi	RNA interference
CPP	Cell-penetrating Peptide
RISC	RNA-Induced Silencing Complex
aiRNA	Asymmetric interfering RNA
DsiRNA	Dicer-substrate siRNA
sisiRNA	Small internally segmented interfering RNA
ARC	Antibody siRNA Conjugate
IGF1	Insulin-like Growth Factor 1
HUVEC	Human Umbilical Vein Endothelial Cells
PSMA	Prostate-specific Membrane Antigen
ASGRP	Asialoglycoprotein receptor
GalNAc	N-acetylgalactosamine
HDL	High Density Lipoprotein
LDL	Low Density Lipoprotein
SR-BI	Scavenger Receptor Class B Member 1
CDM	Carboxylated Dimethyl Maleic acid
NAG	*N*-Acetyl-d-Glucosamine
LCA	Lithocholic Acid
DCA	Docosanoic Acid
PC-DCA	Phosphocholine-DCA
HBFC	sulfosuccinimidyl 4-(p-maleimidophenyl)butyrate
MBS	m-MaleimidoBenzoyl *N*-hydroxySuccinimide
SPDP	Succinimidyl 3-(2-PyridylDithio)Propionate
CDK9	Cyclin-Dependent Kinase 9
MMP2	Matrix MetalloPeptidase 2
LCST	Lower Critical Solution Temperature
SNA	Spherical Nucleic Acid
MN	Magnetic Nanoparticle
QD	Quantum Dot
